# Quality of Life in CAM and Non-CAM Users among Breast Cancer Patients during Chemotherapy in Malaysia

**DOI:** 10.1371/journal.pone.0139952

**Published:** 2015-10-09

**Authors:** Ping Lei Chui, Khatijah Lim Abdullah, Li Ping Wong, Nur Aishah Taib

**Affiliations:** 1 Department of Nursing Science, Faculty of Medicine, University of Malaya, Kuala Lumpur, Malaysia; 2 Department of Social and Preventive Medicine, Faculty of Medicine, University of Malaya, Kuala Lumpur, Malaysia; 3 Department of Surgery, Faculty of Medicine, University of Malaya, Kuala Lumpur, Malaysia; Sudbury Regional Hospital, CANADA

## Abstract

**Background:**

Complementary and alternative medicine (CAM) use has become increasingly popular among patients with cancer. The purposes of this study were to compare the QOL in CAM users and non-CAM users and to determine whether CAM use influences QOL among breast cancer patients during chemotherapy.

**Methodology:**

A cross-sectional survey was conducted at two outpatient chemotherapy centers. A total of 546 patients completed the questionnaires on CAM use. QOL was evaluated based on the European Organization for Research and Treatment of Cancer (EORTC) core quality of life (QLQ-C30) and breast cancer-specific quality of life (QLQ-BR23) questionnaires.

**Results:**

A total of 70.7% of patients were identified as CAM users. There was no significant difference in global health status scores and in all five subscales of the QLQ C30 functional scales between CAM users and non-CAM users. On the QLQ-C30 symptom scales, CAM users (44.96±3.89) had significantly (*p* = 0.01) higher mean scores for financial difficulties than non-CAM users (36.29±4.81). On the QLQ-BR23 functional scales, CAM users reported significantly higher mean scores for sexual enjoyment (6.01±12.84 *vs*. 4.64±12.76, *p* = 0.04) than non-CAM users. On the QLQ-BR23 symptom scales, CAM users reported higher systemic therapy side effects (41.34±2.01 *vs*. 37.22±2.48, *p* = 0.04) and breast symptoms (15.76±2.13 *vs*. 11.08±2.62, *p* = 0.02) than non-CAM users. Multivariate logistic regression analysis indicated that the use of CAM modality was not significantly associated with higher global health status scores (*p* = 0.71).

**Conclusion:**

While the findings indicated that there was no significant difference between users and non-users of CAM in terms of QOL, CAM may be used by health professionals as a surrogate to monitor patients with higher systemic therapy side effects and breast symptoms. Furthermore, given that CAM users reported higher financial burdens (which may have contributed to increased distress), patients should be encouraged to discuss the potential benefits and/or disadvantages of using CAM with their healthcare providers.

## Introduction

Complementary and alternative medicine (CAM) generally refers to a variety of approaches that are not considered as part of Western medicine. At the very core, complementary medicine refers to therapies used together with conventional medicine, while alternative medicine refers to therapies used to substitute conventional medicine, which is generally not recommended [1]. The use of CAM is popular among cancer patients. According to a systematic review of 26 surveys from 13 countries carried out in 1998, the average prevalence of CAM use among adults with cancer was 31.4% [2]. A survey conducted in 2005 across 14 European countries reported that 35.9% of cancer patients used some form of CAM [3]. A recent systematic review and meta-analysis across studies conducted in Australia, Canada, Europe, New Zealand, and the United States highlighted that the combined prevalence for “current use” of CAM in cancer patients was 40% [4]. In Asia, the prevalence of CAM use among cancer patients was found to be higher, ranging from 45% to 98% [5]. In Malaysia, the prevalence of CAM use among overall cancer survivors was approximately 60% [6]. The prevalence of CAM use among breast cancer survivors was estimated to be 64% [7] and 51% [8], while the prevalence of CAM use among breast cancer patients attending follow-up care in a tertiary hospital was 88.3% [9].

Patients opting for CAM seek to restore health and improve quality of life (QOL) [10–11]. QOL is defined by the World Health Organization Quality of Life (WHOQOL) Group as a broad multidimensional concept that usually includes subjective evaluations of both positive and negative aspects of life [12]. With regard to health-related QOL, the Center for Disease Control and Prevention [13] refers to QOL as “a multidimensional concept that includes domains related to physical, mental, emotional, and social functioning.” QOL is therefore regarded as an important aspect in the lives of cancer patients, and assessment of QOL is increasingly being used as a primary outcome measure [14].

In recent years, studies have been conducted on the use of CAM and QOL [15–19]. Despite variations in the design, methodology, and operational definition of CAM, a number of researchers have reported no differences in QOL between CAM users and non-CAM users [15–17]. In contrast to this finding, some evidence points to CAM users having a lower overall QOL than non-CAM users [18–19]. In Malaysia, Farooqui et al. [20] reported that there was no significant difference in overall QOL between users and non-users of three categories of CAM (energy medicine, manipulative body-based therapies, and whole medical system) among cancer patients. However, no prior assessment has been carried out on QOL and CAM use during chemotherapy among patients with breast cancer, when they are considered to be at the most vulnerable period of treatment. The purposes of this study were to compare the QOL in CAM users and non-CAM users and to determine whether CAM use influences QOL among breast cancer patients.

## Materials and Methods

### Design and setting

This is a descriptive cross-sectional design study. The study setting was at two premier outpatient chemotherapy centers: the Hospital Kuala Lumpur (HKL) and the University of Malaya Medical Centre (UMMC) outpatient chemotherapy center. The General Hospital of Kuala Lumpur (or HKL) is a government tertiary referral hospital with 2,302 beds [21] under the Ministry of Health in Malaysia and is one of the largest in Asia. The HKL outpatient chemotherapy center is the first hospital to provide chemotherapy infusion to cancer patients in Malaysia since 1996. It is a national referral center in the field of oncology. The UMMC is a renowned premier teaching hospital with 980 beds under the Ministry of Education in Malaysia [22]. The UMMC outpatient chemotherapy center was established in 1997 and has provided ambulatory chemotherapy services to cancer patients from various parts of the country. CAM services are not part of the standard care and none of the existing health educational leaflets for cancer chemotherapy include information regarding CAM use at these chemotherapy referral centers.

### Population and sampling

Sample size calculation was conducted to establish the required sample size for this study. It was based on an estimated total average population size of 700 new breast cancer cases treated at the HKL and UMMC outpatient chemotherapy centers annually in 2010 and 2011. During the planning phase of this study, little evidence was available concerning CAM use among patients with breast cancer in Malaysia. To establish the sample size of the study, the number of participants necessary to estimate the prevalence of CAM with a 95% confidence interval and 2.0% width margin of error was evaluated. Based on these considerations and an expected 50% prevalence of CAM [8], the necessary number of participants was calculated to be 542. Participant selection was based on the following criteria: patients with breast cancer who had undergone at least one cycle of standard-dose adjuvant chemotherapy and were commencing their subsequent chemotherapy cycle (representing the point at which patients are most likely to anticipate a series of cancer- and chemotherapy-related symptoms and side effects); no previous history of cancer or past receipt of chemotherapy; and at least a basic literacy level in either English, Bahasa Malaysia (Malay language), Mandarin, or Tamil.

### Questionnaire

The questionnaire consisted of three main parts. Part I comprised questions that solicited the demographic, disease, and treatment characteristics of the patients. Part II consisted of questions related to CAM usage. The use of CAM during chemotherapy was assessed by responses to the question: “Which of the following CAMs have you used (at least 4 times) and are currently using while undergoing chemotherapy?” The responses were dichotomous: “Yes” or “No” to CAMs relevant to the local context that were identified based on a literature review [7–8, 23–28] and discussion with a panel of experts.

Part III consisted of items adopted from the European Organization for Research and Treatment of Cancer (EORTC): the EORTC core quality of life (EORTC QLQ-C30) items and EORTC breast cancer-specific quality of life (EORTC QLQ-BR23) items assess breast cancer patients’ QOL. The EORTC QLQ-C30 is a 30-item questionnaire composed of both multi-item scales and single-item measures. These include a global health status or QOL scale (two items), five functional scales (five items on physical functioning, four items on emotional functioning, and two items each on role functioning, cognitive functioning, and social functioning), and nine symptom scales (three items on fatigue, two items each on nausea/vomiting and pain, and one item each on dyspnea, insomnia, appetite loss, constipation, diarrhea, and financial difficulties). The participants were requested to indicate the extent to which they experienced symptoms based on the descriptors: 1 (not at all), 2 (a little), 3 (quite a bit), and 4 (very much). A high score for a functional scale represented a high or healthy level of functioning, a high score for global health status or QOL represented a high QOL, but a high score for a symptom scale or item represented a high level of problems.

The EORTC QLQ-BR23, a breast cancer-specific questionnaire, consists of 23 items on four functional scales (four items on body image, two items on sexual functioning, one item on sexual enjoyment, and one item on perception about the future) and four symptom scales (seven items on systemic therapy side effects, four items on breast symptoms, three items on arm symptoms, and one item on upset by hair loss). The scoring is identical to that of the function and symptom items of the EORTC QLQ-C30. These questionnaires were made available in English, Bahasa Malaysia, Mandarin, and Tamil in order to cater to the multilingual and multi-ethnic target population. The English [29], Bahasa Malaysia [30], Mandarin [31], and Tamil [29] versions of the EORTC QLQ-C30 and EORTC QLQ-BR23 were found to have good internal consistency reliability with Cronbach’s alphas that ranged between 0.70 and 0.87.

### Data collection

The data were collected through a researcher-administered survey, which is less burdensome than the self-administered method. As the sample frame was small, all newly diagnosed breast cancer patients (no previous diagnosis of cancer or chemotherapy prior to this current breast cancer diagnosis) who attended the HKL and UMMC outpatient chemotherapy centers between March 2012 and August 2013 and met the inclusion criteria were invited to participate in the study. The participants were asked to retrospectively recall their CAM use and QOL experienced during the past week (i.e., the third week after the previous chemotherapy). Of the 699 patients approached, 85 patients were excluded from the study because they did not meet the inclusion criteria and 51 refused to participate. Five hundred and sixty-three patients consented to participate in the study. In total, 546 patients completed the questionnaire. The response rate was 88.9% (546 patients completed the questionnaire divided by 614 who met the inclusion criteria). The reasons for non-completion included being too tired (n = 5), having poor physical health (n = 4), being somewhat disinterested (n = 3), declined to answer some of the items (n = 2), and finding the process too burdensome because they were currently involved in another study (n = 3).

The study was registered with the National Medical Research Registry (NMRR-10-111-5204) and was approved by the Ethical Committee of the Medical Research Ethics Committee (MREC), Ministry of Health Malaysia, and the UMMC Medical Ethics Committee (Ref.770.18). All participants were reassured that the confidentiality of their information would be maintained, and informed written consent was obtained from each participant.

### Statistical analysis

Participants were classified as CAM users if they had used or practiced at least one type of CAM at least four times from the start of chemotherapy and were currently using it. Four times is suggested as a minimum marker for commitment in CAM use [32]. CAM was broadly categorized into 3 main groups guided by definitions set by the National Center for Complementary and Integrative Health [1]. These constituted mind-body practices (MBPs), natural products (NPs), and traditional medicine (TM). The MBPs included a large and diverse group of procedures or techniques that were administered or taught by a trained practitioner (aromatherapy, acupuncture, cupping/bekam, exercise, massage, meditation, prayer, reiki, tai chi, therapeutic or healing touch, and yoga) to harmonize communication between mind and body and thereby maintain the patient’s health. The NPs included various dietary supplements (antioxidant capsule/tablet, bird nest, chlorella, cleansing detoxifying diet, ginseng, herbs, jamu, lingzhi, royal jelly, shark cartilage, spirulina, and vitamin and mineral supplements) that were ingested by the patient to maintain or promote their health. The TM included indigenous medicines that were administered by traditional healers, traditional Chinese herbal medicine, ayurvedic/siddha medicine, and homeopathy. CAM users were further categorized as single-modality MBPs, NPs, or TM users. Users of two modalities of CAM were defined as dual-modality (MBP-NP, MBP-TM or NP-TM) users, and those that had used three modalities of CAM were defined as triple-modality MBP-NP-TM users. The baseline characteristics of CAM users and non-users were compared using Pearson’s chi-square test or Fisher’s exact test. Associations with significance levels of less than 0.20 (*p* < 0.20) in the univariate analysis were included in a multivariate logistic regression model to examine the associated characteristics that contributed to CAM use.

The EORTC QLQ-C30 and EORTC QLQ-BR23 items were scored in accordance with the EORTC Scoring Manual [33]. After scoring, all scales were linearly transformed to a 0–100 scale. A general linear model was conducted to examine marginal mean scores of each subscale between CAM users and non-users and between single-modality MBP, dual-modality MBP-NP, and triple-modality MBP-NP-TM users. The results were adjusted for patients’ total symptom burden and demographic, disease, and treatment characteristics. K-means clustering was used to divide CAM users’ global health status scores (range 0–100) into 2 clusters. All statistical analyses were performed using Statistical Package for the Social Sciences Version 18.0 [34]. The statistical assessments were two-sided, and statistical significance was set at an alpha level of 0.05.

## Results

### Demographic, disease, and treatment characteristics of CAM users vs. non-CAM users

Table 1 summarizes the demographic, socio-economic, disease, and treatment characteristics of CAM users and non-CAM users. Three hundred and eighty-six (70.7%) patients reported using some form of CAM, whilst 160 (29.3%) were non-users of CAM. There were significant differences (*p* < 0.001) in education level, average monthly household income, staging of disease, and chemotherapy adherence between CAM users and non-CAM users. The multivariate logistic model indicated that educational level, average monthly household income, staging of disease, and chemotherapy adherence remained significant even after controlling for other variables. The odds of CAM use among patients who postponed their chemotherapy schedules were 5.71 times higher than in patients who adhered to their chemotherapy schedule. The odds of CAM use among patients with average monthly household incomes above RM 3000 were 3.41 times higher than in patients with average monthly household incomes of RM 3000 and below. The odds of CAM use among patients with tertiary education were 2.31 times higher than in patients with primary or lower educational level. The odds of CAM use among patients with advanced-stage breast cancer were 1.86 times higher than in patients with early-stage breast cancer.

**Table 1:** Characteristics of CAM users and non-CAM users (N = 546).

Characteristics	CAM	Non-CAM	*p*-value	Multivariate logistic
	users	users		regression
	n = 386	n = 160		CAM users *vs*.
				non-CAM users
				Adjusted OR
				(95%CI)
**Age**			0.52	
30–39	45(11.7)	13(8.8)		-
40–49	109(28.2)	46(29.8)		-
50–59	167(43.3)	68(42.1)		-
60 and above	65(16.8)	33(19.3)		-
**Ethnicity** ^a^			0.47	
Malay	179(46.4)	67(41.9)		-
Chinese	141(36.5)	66(41.2)		-
Indian	54(14.0)	26(16.2)		-
Others	12(3.1)	1(0.6)		-
**Education level**			<0.001***	
Primary/lower	68(17.6)	51(31.9)		1
Secondary school	203(52.6)	88(55.0)		1.42(0.90–2.24)
Tertiary	115(29.8)	21(13.1)		2.31(1.22–4.47)*
**Marital status**			0.63	
Single	44(11.4)	16(10.0)		-
Ever married	342(88.6)	144(90.0)		-
**Household income/month**			<0.001***	
≤RM 3000	253(65.5)	140(87.5)		1
>RM 3000	133(34.5)	20(12.5)		3.41(1.92–6.02)***
**Staging of disease**			0.03*	
Early	228(59.1)	110(68.8)		1
Advanced	158(40.9)	50(31.2)		1.86(1.23–2.82)**
**Menopausal status**			0.13	
Pre menopause	184(47.7)	65(40.6)		1
Post menopause	202(52.3)	95(59.4)		1.00(0.66–1.52)
**Chemotherapy regimen**			0.16	
Docetaxel	91(23.6)	29(18.1)		1
FEC/FAC/CMF/AC^b^	295(76.4)	131(81.9)		0.86(0.53–1.41)
**Chemotherapy cycle**			0.66	
2, 3 and 4	258(66.8)	110(68.8)		-
5 and 6	128(33.2)	50(31.2)		-
**Chemotherapy adherence** ^c^			<0.001***	
On schedule	345(89.4)	156(97.5)		1
Postponed	41(10.6)	4(2.5)		5.71(1.97–16.53)**
**Total symptom burden**			0.73	
**score** ^d^				
High (54–130)	92 (23.8)	36(22.5)		-
Low (13–53)	294(76.2)	124(77.5)		-

^a^ Other ethnic groups were excluded from the analysis

^b^ FEC (5-fluorouracil/5-FU, epirubicin and cyclophosphamide), FAC (5-FU, doxorubicin and cyclophosphamide), CMF (cyclophosphamide, methotrexate and 5FU), AC (doxorubicin and cyclophosphamide)

^c^ Fisher’s exact test

^d^ An adaptation of the 16-item Side Effect Burden Scale [35] was used to measure patients’ symptom burden. K-means clustering was used to divide patients into 2 clusters of high and low total symptom burden.

* Significance level at *p* < 0.05

**Significance level at *p* < 0.01

***Significance level at *p* < 0.001

Among CAM users, most of them were users of multiple CAM modalities (Table 2). The three categories of CAM used by more than 10% of the patients who employed at least one CAM modality were: 1) MBP (single-modality), 2) MBP-NP (dual-modality), and 3) MBP-NP-TM (triple-modality).

**Table 2:** Pattern of CAM usage (N = 386).

CAM	Single-	Dual-	Dual-	Single-	Dual-	Single-	Triple-
usage	modality	modality	modality	modality	modality	modality	modality
	MBP	MBP-NP	MBP-TM	NP	NP-TM	TM	MBP-NP-
	users	users	users	users	users	users	TM
							users
				n(%)			
	50 (13.0)	180(46.6)	24 (6.2)	27 (7.0)	4 (1.0)	13 (3.4)	88 (22.8)

### Quality of life (EORTC QLQ-C30) scores between CAM users and non-CAM users

After adjusting for total symptom burden and demographic, disease, and treatment characteristics, there were no significant differences between CAM users and non-CAM users in global health status scores and on all functional scales (Table 3). There were no statistical differences for the symptom scales either, except for financial difficulties, where CAM users (44.96±3.89) had significantly (*p* = 0.01) higher marginal mean scores for financial difficulties than non-CAM users (36.29±4.81).

**Table 3:** Quality of life (EORTC QLQ-C30) scores between CAM users and non-CAM users ^a^.

	All (N = 546)		
	Non-CAM users	CAM users	*p*-value
	n = 160	n = 386	
EORTC QLQ-C30	Mean±SE	Mean±SE	
**Global health status**	64.52±2.94	62.95±2.38	0.40
**Functional scales**			
Cognitive functioning	77.88±2.93	76.72±2.37	0.53
Physical functioning	81.84±2.21	79.97±1.79	0.18
Emotional functioning	74.62±2.95	73.85±2.39	0.68
Role functioning	72.89±3.87	70.34±3.13	0.30
Social functioning	63.13±3.94	61.35±3.19	0.48
**Symptom scales**			
Fatigue	43.36±2.81	42.96±2.27	0.82
Financial difficulties	36.29±4.81	44.96±3.89	0.01*
Appetite loss	29.71±4.16	29.58±3.37	0.96
Insomnia	32.94±3.89	33.95±3.15	0.68
Pain	23.19±2.88	26.31±2.33	0.09
Nausea & vomiting	22.26±3.20	24.88±2.59	0.20
Constipation	37.70±4.22	33.43±3.42	0.11
Diarrhoea	21.42±3.74	22.47±3.03	0.66
Dyspnoea	11.97±2.91	12.11±2.36	0.94

^a^ Adjusted for total symptom burden, and demographic, disease and treatment characteristics

*Significance level at *p* < 0.05

CAM modality used by less than 10% of CAM users was excluded from the analysis. Among CAM modalities used by more than 10% of the CAM users (N = 318) (Table 4), there were no significant differences between single-modality MBP users, dual-modality MBP-NP users, and triple-modality MBP-NP-TM users in global health status scores and all symptom scales. On the functional scales, a significant difference in cognitive and emotional functioning mean scores was evident across single-modality MBP, dual-modality MBP-NP, and triple-modality MBP-NP-TM users (*p* = 0.01). The post hoc analysis (Bonferroni) confirmed that the difference in cognitive functioning between single-modality MBP and dual-modality MBP-NP users was significant (*p =* 0.01). The difference in cognitive functioning between single-modality MBP and triple-modality MBP-NP-TM users was significant (*p* = 0.04). The difference in emotional functioning between single-modality MBP and dual-modality MBP-NP users was significant (*p =* 0.01).

**Table 4:** Quality of life (EORTC QLQ-C30) scores among CAM users^a^.

	CAM user (N = 318)			
	Single-	Dual-modality	Triple-	
	modality	MBP-NP	modality	*p*-value
	MBP		MBP-NP-TM	
	n = 50	n = 180	n = 88	
EORTC QLQ-C30	Mean±SE	Mean±SE	Mean±SE	
**Global health status**	60.17±4.10	63.80±3.04	64.83±3.82	0.42
**Functional scales**				
Cognitive functioning	68.58±3.98	78.29±2.95	77.56±3.70	0.01*
Physical functioning	74.80±3.12	80.38±2.31	78.23±2.89	0.08
Emotional functioning	65.08±4.63	75.46±2.99	73.31±3.74	0.01*
Role functioning	68.74±5.67	72.22±4.21	64.18±5.27	0.08
Social functioning	54.49±5.35	62.69±3.97	65.55±4.97	0.06
**Symptom scales**				
Fatigue	49.04±3.87	44.56±2.87	41.74±3.59	0.11
Financial difficulties	46.98±6.56	44.67±4.87	34.73±6.10	0.05
Appetite loss	32.83±5.89	33.47±4.37	40.44±5.48	0.19
Insomnia	27.71±6.13	33.33±4.03	30.29±5.05	0.39
Pain	33.08±3.64	28.37±2.69	25.98±3.38	0.09
Nausea & vomiting	23.52±4.10	27.03±3.04	26.78±3.82	0.55
Constipation	35.10±4.94	27.24±3.66	30.97±4.59	0.13
Diarrhoea	23.67±5.01	25.25±3.72	26.72±4.66	0.78
Dyspnoea	16.90±4.13	1.29±3.07	13.82±3.84	0.23

^a^ Adjusted for total symptom burden, and demographic, disease and treatment characteristics

*Significance level at *p* < 0.05

### Breast cancer-specific quality of life (EORTC QLQ-BR23) scores between CAM users and non-CAM users

Table 5 shows the marginal mean scores for each of the EORTC QLQ-BR23 scales. CAM users (6.01±12.84) had significantly (*p* = 0.04) higher sexual enjoyment mean scores than non-CAM users (4.64±12.76). The sexual enjoyment scores of CAM users and non-CAM users were based on 125 and 58 sexually active patients, respectively. As for symptom scales, CAM users (41.34±2.01) reported significantly (*p* = 0.04) higher systemic therapy side effect mean scores than non-CAM users (37.22±2.48). Likewise, CAM users (15.76±2.13) reported significantly (*p* = 0.02) higher breast symptom mean scores than non-CAM users (11.08±2.62).

**Table 5:** Breast cancer-specific quality of life (EORTC QLQ-BR23) scores between CAM users and non-CAM users ^a^.

	All (N = 546)		
	Non-CAM users	CAM users	*p*-value
	n = 160	n = 386	
EORTC QLQ-BR23	Mean±SE	Mean±SE	
**Functional scales**			
Body image	75.59±4.63	73.25±3.75	0.43
Future perspective	50.63±5.24	48.73±4.25	0.57
Sexual enjoyment	4.64±12.76^b^	6.01±12.84^c^	0.04*
Sexual functioning	12.23±3.18	9.00±2.58	0.11
**Symptom scales**			
Upset by hair loss	36.29±6.00	35.63±4.86	0.86
Systemic therapy side effects	37.22±2.48	41.34±2.01	0.04*
Arm symptoms	16.28±2.87	18.97±2.33	0.14
Breast symptoms	11.08±2.62	15.76±2.13	0.02*

^a^ Adjusted for total symptom burden, and demographic, disease and treatment characteristics

^b^ 58 sexually active patients responded

^c^ 125 sexually active patients responded

*Significance level at *p* < 0.05

Among CAM users (Table 6), a significant difference (*p* < 0.05) in body image and future perspective means scores was evident across single-modality MBP, dual-modality MBP-NP, and triple-modality MBP-NP-TM users. On the symptom scales, significant differences in upset by hair loss and systemic therapy side effect mean scores were evident across single-modality MBP, dual-modality MBP-NP, and triple-modality MBP-NP-TM users (*p* < 0.05). Post hoc analysis (Bonferroni) showed that the differences in body image between single-modality MBP and dual-modality MBP-NP, and between single-modality MBP and triple-modality MBP-NP-TM users, were significant (*p* < 0.001). On future perspective, the differences between single-modality MBP and dual-modality MBP-NP, and between single-modality MBP and triple-modality MBP-NP-TM users, were significant (*p* < 0.001). On symptom scales, the difference in upset by hair loss between single-modality MBP and dual-modality MBP-NP users was significant (*p* = 0.003). The differences in systemic therapy between single-modality MBP and dual-modality MBP-NP, and between single-modality MBP and triple-modality MBP-NP-TM users, were significant (*p* < 0.001).

**Table 6:** Breast cancer-specific quality of life (EORTC QLQ-BR23) scores among CAM users^a^.

	CAM users (N = 318)			
	Single-	Dual-	Triple	*p-*value
	modality	modality	modality	
	MBP	MBP-NP	MBP-NP-TM	
	n = 50	n = 180	n = 88	
EORTC QLQ-BR23	Mean±SE	Mean±SE	Mean±SE	
**Functional scales**				
Body image	54.47±5.89	75.82±4.38	76.31±5.48	<0.001*
Future perspective	37.42±6.04	53.64±4.48	55.47±5.61	0.001*
Sexual enjoyment	4.58±9.31^b^	19.61±7.89^c^	9.39±9.28^d^	0.10
Sexual functioning	11.62±4.27	11.25±3.17	11.61±3.97	0.99
**Symptom scales**				
Upset by hair loss	49.10±7.09	34.36±5.26	39.10±6.59	0.04*
Systemic therapy side	45.27±2.53	40.03±1.88	39.47±2.35	0.02*
effects				
Arm symptoms	16.94±3.79	15.68±2.81	18.51±3.52	0.54
Breast symptoms	16.68±3.10	16.51±2.30	20.59±2.88	0.14

^a^ Adjusted for total symptom burden, and demographic, disease and treatment characteristics

^b^ A total of 20 MBP users reported as being sexually active answered these

^c^ A total of 51 MBP-NP users reported as being sexually active answered these

^d^ A total of 39 MBP-NP-TM users reported as being sexually active answered these

*Significance level at *p* < 0.05

### Factors associated with mean global health status score

Table 7 shows the univariate and multivariate analyses of factors associated with global health status (QOL) in two subgroups identified by cluster analysis. Other ethnic groups and CAM modality used by less than 10% of CAM users were excluded from the analysis due to small sample size. In the 2-cluster classifications of global health status scores, Cluster I consisted of 182 participants who had high global health status scores ranging from 66 to 100 (80.27±0.97). Cluster II consisted of 126 participants with low global health status scores ranging from 0 to 58 (41.20±1.17). Factors with significance levels lower than 0.20 (*p* < 0.20) in the univariate analysis between high and low global health status clusters were included in the multivariate analysis.

**Table 7:** Factors associated with high and low global health status (QOL) (N = 308)^a^ ^,^ ^b^.

Characteristics	Global health status		*p*-value	Multivariate logistic
	score			regression
	High	Low		(High *vs*. Low)
	n = 182	n = 126		
	n (%)	n (%)		Adjusted OR (95%CI)
**Age**			<0.001	
30–39	27(14.8)	8(6.3)		1.39(0.35–5.54)
40–49	38(20.9)	36(28.6)		1.48(0.48–4.52)
50–59	92(50.5)	47(37.3)		1.94(0.85–4.39)
60 and above	25(23.7)	35(27.8)		1
**Ethnicity** ^a^			<0.001	
Malay	100(54.9)	51(40.5)		9.19(3.71–22.71) **
Chinese	66(36.3)	38(30.2)		3.47(1.48–8.11) **
Indian	16(8.8)	37(29.4)		1
**Education level**			0.05	
Primary/lower	27(14.8)	28(22.2)		1
Secondary	85(46.7)	65(51.6)		1.37(0.59–3.16)
Tertiary	70(38.5)	33(26.2)		4.29(1.58–11.61) **
**Marital status**			0.05	
Single	26(14.3)	9(7.1)		3.11(1.10–8.82)*
Ever married	156(85.7)	117(92.9)		1
**Household’s income**			0.18	
≤RM 3000	106(58.2)	3(65.9)		2.52(1.24–5.12) *
>RM 3000	76(41.8)	43(34.1)		1
**Staging of disease**			0.28	
Early	100(54.9)	77(61.1)		**-**
Advanced	82(45.1)	49(38.9)		**-**
**Menopausal status**			0.09	
Pre menopausal	87(47.8)	48(38.1)		1.31(0.64–2.83)
Post menopausal	95(52.2)	78(61.9)		1
**Chemotherapy regimen**			0.04	
Docetaxel	52(28.6)	23(18.3)		1.47(0.72–2.99)
FEC/FAC/CMF/AC	130(71.4)	103(81.7)		1
**Chemotherapy cycle**			0.73	
2, 3 or 4	115(63.2)	82(65.1)		**-**
5 or 6	67(36.8)	44(34.9)		**-**
**Chemo adherence**			0.10	
Postponed	20(11.0)	7(5.6)		5.37(1.36–21.11) *
On schedule	162(89.0)	119(94.4)		1
**CAM modality** ^b^			0.71	
MBP	30(16.5)	18(14.3)		**-**
MBP-NP	103(56.6)	69(54.8)		**-**
MBP-NP-TM	49(26.9)	39(31.0)		**-**
**Total symptom burden**			<0.001	
**score**				
High (54–130)	11(6.0)	58(46.0)		1
Low (13–53)	171(94.0)	68(54.0)		28.89(11.53–72.41) ***

^a^ Other ethnic groups were excluded in the analysis

^b^ CAM modality used by less than 10% of CAM users was excluded from the analysis

* Significance level at *p* < 0.05

**Significance level at *p* < 0.01

***Significance level at *p* < 0.001

The use of CAM modality was not significantly associated with high global health status scores (*p* = 0.71). Multivariate logistic regression analysis indicated that the odds of high global health status among patients with low total symptom burden were 28.89 times higher than in patients with high total symptom burden. The odds of high global health status among Malay and Chinese patients were 9.19 and 3.47 times higher than in Indian patients, respectively. The odds of having high global health status among patients who had delayed chemotherapy cycles at some point were 5.37 times higher than in patients who had completed their chemotherapy on schedule.

The odds of high global health status among patients with tertiary education were 4.29 times greater than in patients with primary or lower educational level. The odds of high global health status among single patients were 3.11 times greater than in married patients. The odds of having high global health status among patients with average monthly household incomes of RM 3000 or below were 2.52 times higher than in patients with household incomes above RM 3000. In the test of the multivariate model’s goodness of fit, the Chi-square value for the Hosmer-Lemeshow test was 11.93, corresponding to a significance level of 0.15 (*p* > 0.05), implying consistency with a good fit.

## Discussion

The results of the present study suggest that CAM use is common among Malaysian breast cancer patients who are undergoing chemotherapy. CAM users in this study were less likely to receive their chemotherapy regimen on schedule. This is consistent with findings by Al-Naggar et al. who found that 16.4% of cancer patients stopped standard treatment while using CAM at two referral hospitals in Malaysia [36]. CAM users in this study had a higher monthly household income, held tertiary education, and were at an advanced stage of cancer. The findings were consistent with previous studies where CAM users had higher education [3,8,36–38], higher household income [3,39], and advanced cancer stage [40], all of which are factors which have been documented as significantly associated with the use of CAM. Patients with higher education and a higher economic status may be more likely to have the financial means to search for other therapies to cope with the disease and treatment effects [41]. Likewise, patients at advanced stages of cancer may experience higher stress and lower immunity. Thus, they may be more likely to use CAM for stress reduction and for strengthening their immune system [42].

It is worth noting that there was no significant difference in global health status between CAM users and non-CAM users, and between single-modality MBP, dual-modality MBP-NP, and triple-modality MBP-NP-TM users in the present study. This finding is consistent with a study conducted by Farooqui et al. [20] in Malaysia and with Can et al.’s study [15] that reported no differences in QOL between CAM users and non-CAM users in Turkey. Similarly, Kang et al. [16] and Tautz et al. [17] reported that the global QOL between CAM users and non-users was similar among patients with breast cancer in Korea and Germany, respectively. However, several previous studies [18–19] found that CAM users had a lower QOL than non-CAM users. It is possible that breast cancer patients who are most attracted to CAM may be seeking relief from a perceived low QOL [19]. However, the current study revealed a higher global health status score than the previous study in Malaysia [20]. This disparity may be due to different patient populations and cancer types. The current study recruited female patients with breast cancer, while Farooqui et al. sampled male and female patients with different types of cancer [20].

This study also found no significant differences in QOL on all functional subscales between CAM users and non-CAM users. CAM users, however, appeared to experience more financial difficulties than non-CAM users. A possible explanation would be that this study was conducted in public hospitals in Malaysia, which are usually visited by middle to low income patients. The financial burden of cancer treatment and additional cost of CAM may be the reason for financial difficulties. This is in line with Farooqui et al.’s [20] findings, who found no significant difference in QOL on all functional subscales between CAM users and non-CAM users in Malaysia. They also reported that CAM users faced more financial difficulties and insomnia on symptom scales than non-CAM users. Further findings on breast cancer-specific QOL assessment show that CAM users in this study suffered more from systemic therapy side effects and breast symptoms than non-CAM users. It is possible that breast cancer patients who are most attracted to CAM seek relief from the perceived combination of systemic therapy side effects and breast symptoms during chemotherapy.

An exception to this was that CAM users appeared to have higher sexual enjoyment than non-CAM users. This result differs from Tautz et al. [17], who found that CAM users exhibited a lower QOL on emotional, cognitive, and social functioning subscales and suffered more from side effects of systemic therapies than non-CAM users. Kang et al. [16] found no significant differences in functional and symptom subscale scores between CAM users and non-CAM users. Changes to sexual well-being can be one of the most problematic aspects of life after breast cancer. The findings that CAM users appeared to have higher sexual enjoyment than non-CAM users in this study should be interpreted with caution because they were based on a small proportion of sexually active patients.

In particular, dual-modality MBP-NP users were found to have a better QOL in cognitive and emotional functioning, while triple-modality MBP-NP-TM users appeared to have a better body image and future perspective. Dual-modality MBP-NP users and triple-modality NP-MBP-TM users also reported being less upset by hair loss and systemic therapy side effects, respectively. Of greater concern here is that consumption of most of the NPs and application of TM concurrent with chemotherapy should be done with caution, given that queries related to their safety and benefits remain unanswered. Further study with greater focus on specific MBP, NP, and TM use is therefore recommended.

The key finding of the study is that CAM modality use was not significantly associated with QOL after controlling for other variables. However, the total symptom burden was significantly associated with QOL. This is in line with Yong et al. [43] who reported that QOL correlated negatively with symptom burden in a study among patients with end-stage renal disease. Malay patients were more likely to have a better QOL than Indian patients. Further study is required to establish this in the multi-ethnic and multicultural society in Malaysia. In this study, it is interesting to note that CAM users who were less likely to receive their chemotherapy regimen on schedule were more likely to have a better QOL. This is contrary to the notion that neutropenia-induced delays in chemotherapy diminished the psychological well-being of patients and negatively impacted patients’ QOL [44]. This finding warrants further study on the association between neutropenia-induced delays in chemotherapy, CAM use, and QOL. Nevertheless, the findings should be interpreted with caution because only a small proportion of patients had delayed their chemotherapy schedule in this study.

Single patients in this study were more likely to have a better QOL than married patients. This is in line with a cross-sectional study conducted by Jassim and Whitford [45] on the QOL of Bahrainian women with breast cancer. However, the current study differs from several previous studies [46–48], which showed that married patients with breast cancer had better QOL. One reason for the low QOL among married breast cancer patients in this study could be that the majority of patients were Malays who were fearful of being rejected by their partners or losing their husbands to other women, as Muslims can legally have up to four wives [45]. Another possible explanation is that social support can be viewed as both a positive and negative factor. Thus, it can be either an encouragement to strengthen the patient’s will to fight for life or a source of distress when the family overly shows concern, attention, and love [49].

Patients with higher education levels were more likely to have a better QOL in this study. The exception is that patients with a lower average monthly household income were more likely to have a better QOL. Previous studies have reported higher education level and income as being positively associated with QOL [47,50]. Patients with higher education and more income were more likely to have better access to information and resources for problem solving as well as better coping skills, which might explain better QOL among women with higher socioeconomic status. Moreover, patients with higher education are more likely to assess and understand information on breast cancer and its management, which would be helpful to improve QOL [50]. In addition, according to Ashing-Giwa and Lim [51], physicians spend more time with affluent and educated patients than with financially and educationally deprived patients who actually need more attention and care. This may also be one of the reasons why a higher educational level and income could influence the QOL of breast cancer patients.

The findings that patients with lower average monthly household income were more likely to have better QOL in this study are novel and make comparison difficult. However, Pinar, Salepci, and Afsar reported that financial status had no effect on the QOL of Turkish patients with cancer [52]. Other studies have reported higher income as being positively associated with QOL [45,47,50]. This inconsistency could be due to cultural factors characterizing the different populations. These contrasting findings show that there is some value in conducting targeted prospective longitudinal research on this matter.

Although this study has presented many insightful and constructive findings that contribute to an overall understanding of CAM use and QOL, it also has several limitations. First, the data were collected from patients on their chemotherapy appointment day (21 days post chemotherapy). Responses, therefore, may have been subject to recall bias. To mitigate the potential for recall bias, patients were asked to evaluate the QOL that they experienced over the past 7 days rather than the previous 21 days. Second, as the majority of the patients were multiple CAM modality users, further analysis on single-modality CAM users was limited due to a small number of cases. A larger-scale study that includes more participants is needed to provide more precise findings. Third, among non-CAM users, there may have patients who could have used CAM less than four times, and this could have affected the findings. Despite these limitations, this study is the first to examine the QOL in CAM and non-CAM users among breast cancer patients during chemotherapy in Malaysia.

## Conclusion

This study showed that there were no significant differences in QOL between CAM users and non-CAM users, and between single-modality MBP, dual-modality MBP-NP, and triple-modality MBP-NP-TM users. CAM modality use is not a significant correlate for QOL; however, total symptom burden is the strongest correlate after adjusting for other variables. Given that CAM users were more likely to have a higher level of financial difficulty and greater systemic side effects and breast symptoms, health-care providers should pay special attention to CAM users and arrange support for them from appropriate sources. In conclusion, this study has advanced the current understanding of CAM use and QOL among patients with breast cancer during chemotherapy. In particular, the categorization of MBP, NP, and TM as separate modalities may help to better define CAM users and shed light on future research on specific CAM modality users.
